# Enhancing Type 1 Diabetes Polygenic Risk Prediction Through Neural Networks and Entropy-Derived Insights

**DOI:** 10.3390/ijms27072966

**Published:** 2026-03-25

**Authors:** Antonio Nadal-Martínez, Guillermo Pérez-Solero, Sandra Ferreiro López, Jorge Blom-Dahl, Eduard Montanya, Marta Alonso-Bernáldez, Moises Shabot, Christian Binsch, Lukasz Szczerbinski, Adam Kretowski, Julián Nevado, Pablo Lapunzina, Robert Wagner, Jair Tenorio-Castano

**Affiliations:** 1 Soft Computing, Image Processing and Aggregation (SCOPIA) Research Group, University of the Balearic Islands (UIB), 07122 Palma, Spain; 2 Health Research Institute of the Balearic Islands (IdISBa), 07010 Palma, Spain; 3 Artificial Intelligence Research Institute of the UIB (IAIB), University of the Balearic Islands, 07122 Palma, Spain; 4 Research Department Unit, N-GENE, Carretera Betlem, s/n, Colonia de Sant Pere, 07579 Arta, Spain; morgan.monzon@n-gene.ai (G.P.-S.); darwin@adntro.com (S.F.L.); fireblom@adntro.com (J.B.-D.); nettie.stevens@n-gene.ai (M.A.-B.); moises.shabot@n-gene.ai (M.S.); 5 Bellvitge Biomedical Research Institute (IDIBELL), Hospital Universitari de Bellvitge, University of Barcelona, 08907 Barcelona, Spain; montanya@ub.edu; 6 Centro de Investigación Biomédica en Red de Diabetes y Enfermedades Metabólicas Asociadas (CIBERDEM), Instituto de Salud Carlos III, 28029 Madrid, Spain; 7 Department of Endocrinology and Diabetology, Medical Faculty and University Hospital, Heinrich Heine University, 40225 Düsseldorf, Germany; christian.binsch@ddz.de (C.B.); robert.wagner@ddz.de (R.W.); 8 Institute for Clinical Diabetology, German Diabetes Center (DDZ), Leibniz Institute for Diabetes Research at Heinrich Heine University, 40225 Düsseldorf, Germany; 9 German Center for Diabetes Research, München-Neuherberg, 85764 Neuherberg, Germany; 10 Center for Genomic Medicine, Massachusetts General Hospital, Boston, MA 02114, USA; lukasz.szczerbinski@umb.edu.pl; 11 Diabetes Unit, Department of Medicine, Massachusetts General Hospital, Boston, MA 02114, USA; 12 Programs in Metabolism and Medical & Population Genetics, Broad Institute, Cambridge, MA 02142, USA; 13 Department of Endocrinology, Diabetology and Internal Medicine, Medical University of Bialystok, 15-174 Bialystok, Poland; adam.kretowski@umb.edu.pl; 14 Clinical Research Centre, Medical University of Bialystok, 15-174 Bialystok, Poland; 15 Centre for Digital Medicine, Medical University of Bialystok, 15-174 Bialystok, Poland; 16 Instituto de Genética Médica y Molecular (INGEMM), Instituto de Investigación del Hospital Universitario La Paz (IdiPaz), Hospital Universitario La Paz, 28046 Madrid, Spain; jnevadobl@gmail.com (J.N.); plapunzina@gmail.com (P.L.); 17 CIBERER, Centro de Investigación Biomédica en Red de Enfermedades Raras, Instituto de Salud Carlos III, 28029 Madrid, Spain; 18 ERN-ITHACA, European Reference Network on Rare Malformations Syndromes, Intellectual and Other Neuro-Developmental Disorders, 75019 Paris, France; 19 Centro Universitario HM Hospitales de Ciencias de la Salud (CUHMED), Universidad Camilo José Cela, 28660 Madrid, Spain

**Keywords:** type 1 diabetes, polygenic risk score, PRS, neural network, entropy, risk stratification, UK Biobank, machine learning, genomic medicine, newborn screening

## Abstract

Type 1 diabetes (T1D) is an autoimmune disease with a strong genetic component (~70% heritability). Early identification of individuals at risk is crucial for early intervention or risk assessment. Although polygenic risk scores (PRS) have shown promise in risk assessment, most current approaches remain constrained by linear assumptions and limited generalizability. We aimed to develop a neural network-driven classifier using T1D-associated single nucleotide polymorphisms (SNPs). In addition, we explored the inclusion of an entropy-derived feature as a complementary variable, representing the degree of genetic variability within an individual’s genotype profile across the 67 T1D-associated SNPs, to evaluate its potential additive contribution to the model performance. We analyzed genotype data from 11,909 individuals in the UK BioBank (546 T1D cases and 11,363 controls). Sixty-seven well-known SNPs associated with T1D were utilized as inputs to the model, using two distinct allele-encoding strategies. A feed-forward neural network was evaluated under varying case–control ratios through five-fold cross-validation. Performance was assessed using the area under the receiver operating characteristic curve (AUC) on a held-out test set and on an external European cohort as a validation cohort. Across five-fold cross-validation, the best configuration achieved a median AUC of 0.903. On the held-out UK Biobank test set, the model generalized well, with an AUC of 0.8889 (95% CI: 0.8516–0.9262). A probability-based risk framework, constructed using five risk groups (“very low”, “low”, “intermediate”, “high”, and “very high” risk), yielded a negative predictive value (NPV) of 98.9% for the “very low” risk group and a Positive Predicted Value (PPV) of 61.9% with a specificity of 97.3% for the “very high” risk group, assuming a 10% T1D prevalence. External validation in the German Diabetes Study reproduced clear case–control separation; for individuals with recent onset diabetes and glutamic acid decarboxylase antibodies (GADA+) vs. controls, specificity reached 91.9% in the “high” risk group (PPV of 94.3%) and 97.6% in the “very high” risk group (PPV of 95.7%). The proposed neural network reliably predicts T1D genetic risk using a compact SNP panel of 67 SNPs and maintains accuracy in both internal and external European cohorts. Its probabilistic output enables clinically interpretable risk thresholds, while entropy features contributed modestly to performance. These results demonstrate that a neural network-based approach achieves discriminative performance that is comparable to established T1D genetic risk models, while offering flexible probability-based risk stratification and architectural extensibility for future integration of additional features.

## 1. Introduction

Type 1 diabetes (T1D) is a chronic autoimmune disorder characterized by the immune-mediated destruction of pancreatic β-cells, resulting in lifelong dependence on exogenous insulin therapy. Although traditionally considered a pediatric condition, recent evidence shows that T1D occurs across the lifespan, with frequent diagnostic ambiguity in adults due to clinical overlap with type 2 diabetes (T2D) [1]. T1D pathogenesis starts in early life, with β-cell autoantibodies (insulin (IAA), glutamic acid decarboxylase antibody (GADA), islet antigen-2 (IA-2) and zinc transporter 8 (ZnT8).

The presence of multiple islet autoantibodies is a near-certain predictor of progression to clinical disease, with up to 70% of children progressing to overt diabetes within a decade of seroconversion [2]. Genetically, the strongest risk for T1D is conferred by specific HLA class II haplotypes, particularly HLA-DR3-DQ2 and HLA-DR4-DQ8, which account for over 90% of cases in some populations [3]. Non-HLA loci, including variants in INS, PTPN22, and IL2RA, also contribute to genetic disease susceptibility [4], and genome-wide association studies (GWAS) have now identified over 60 loci involved in T1D genomic risk [5]. Importantly, genetic risk is not confined to familial cases; indeed, most children diagnosed with T1D do not have a first-degree relative with the disease [6].

Recent advances in immunomodulatory therapy have opened new pathways for personalized intervention. The FDA’s approval of teplizumab, an anti-CD3 monoclonal antibody, for delaying the clinical onset of T1D in high-risk individuals marks the first immunotherapy approved for pre-symptomatic disease [7]. This development highlights a shift toward an etiological treatment paradigm that aims to preserve β-cell function during the early stages of autoimmunity [8].

Primary prevention strategies, though historically limited by unclear environmental triggers, are being revitalized through population screening initiatives and genetic risk stratification models.

Given the preclinical nature of T1D, early identification of individuals at high risk has become a major goal for primary prevention strategies. Longitudinal studies such as TEDDY [9] and GPPAD [6] have demonstrated the feasibility of genetic risk screening in infancy by using polygenic risk scores (PRS), which integrate both HLA haplotyping and non-HLA SNPs analysis to predict the likelihood of seroconversion to autoantibody positivity and subsequent diabetes [4,5]. Despite advances in genetic prediction, the clinical classification of diabetes remains challenging, particularly in adults, where T1D is frequently misdiagnosed as T2D. Studies have shown that up to 40% of cases may be miscoded, misclassified, or misdiagnosed in primary care records, potentially compromising treatment, research efforts, and patient management [9]. Moreover, racial and ethnic disparities in T1D incidence, outcomes, and glycemic control have been well-documented. African American youth, for instance, experience the substantial burden of T1D alongside T2D, often presenting with poorer metabolic profiles and higher A1C levels, which are associated with increased risk of medical complications [10].

These findings underscore the serious need for robust, ancestry-aware diagnostic tools that leverage both clinical and genomic data to improve the early detection and subtype classification of diabetes, even before the first symptoms appear.

Recent advances in the development and refinement of genetic risk scores (GRSs) for T1D have highlighted the importance of ancestry-aware modeling and cross-population validation, as summarized in the following related studies.

Onengut-Gumuscu et al. [11] addressed the underrepresentation of individuals of African ancestry in genetic studies of T1D by analyzing ImmunoChip SNP data from over 1000 African-ancestry T1D cases and nearly 3000 controls. They have developed an ancestry-specific genetic risk score that incorporated both novel and well-known loci, including African-specific HLA haplotypes and non-HLA SNPs, such as those in the Insulin (*INS*) and Gasdermin-B (*GSDMB*) genes. Their African-specific GRS significantly outperformed a European-derived GRS in African-ancestry samples, achieving an area under the curve (AUC) of 0.871 compared to 0.798 when applying the European-derived GRS to African-ancestry individuals. These findings emphasize the decisive importance of population-specific polygenic risk modeling to enhance the prediction accuracy and guide immune monitoring interventions and screening in diverse populations.

Building upon this, Sharp et al. [12] introduced an improved GRS termed T1D GRS2, specifically designed to enhance the prediction of T1D, with a focus on newborn screening and on the discrimination of T1D at incident diagnosis (particularly from T2D). T1D GRS2 incorporates 67 SNPs, including refined tagging of 14 HLA DR-DQ haplotypes, their interactions, and 32 non-HLA loci. This enhanced model demonstrated a significantly superior discriminative performance, with an AUC of 0.927 in the T1DGC cohort and 0.921 in the UK Biobank validation set. Notably, T1D GRS2 showed an improved ability to differentiate T1D from type 2 diabetes and to identify newborns with increased risk, offering a practical and cost-effective solution for early diagnosis and intervention.

Qu et al. [13] demonstrated that T1D-GRS2 effectively predicts T1D in African American (AUC 0.807) and European American (AUC 0.823) children, with improved performance after adding four African-specific SNPs (AUC 0.826 and 0.839), supporting trans-ethnic calibration. Oram et al. [14] further validated GRS2 in diverse youth, outperforming earlier 30-SNP models, especially in Hispanic and Black individuals, and enabling accurate classification of ambiguous or autoantibody-negative diabetes when combined with a T2D-GRS. More recently, Luckett et al. [15] introduced GRS2x, a standardized and ancestry-aware version incorporating the imputation of missing variants, achieving high predictive accuracy driven mainly by HLA class II effects (AUC up to 0.90) and robust generalization to multiethnic cohorts (AUC ~0.86–0.93), thus enabling scalable early-life genetic risk stratification.

In this study, we explore the use of SNP-based models through a neural network-driven polygenic risk modeling to identify individuals who are at risk for T1D, with the broader goal of enhancing diagnostic accuracy and informing targeted preventive strategies.

## 2. Results

### 2.1. Feed-Forward Neural Network Cross-Validation

We have evaluated the neural network model across different class ratios: 1:1, 1:2 and 1:3 (case:control), as well as using the full unbalanced dataset. The results of the cross-validated experiments and their generalization to the test set are shown in Table 1. As shown in Table 1, the best-performing model achieved a mean AUC of 0.903, using a 1:3 case-to-control ratio and effect-allele-count encoding.

Notably, β-weighted encoding under the 1:3 class ratio showed reduced cross-validation performance compared to raw allele-count encoding. One possible explanation is that externally derived β coefficients, while informative in linear models such as GRS2, may introduce fixed weighting assumptions that interact suboptimally with a moderate class imbalance in nonlinear architectures. In contrast, raw allele encoding allows for the neural network to learn feature importance dynamically during training, potentially providing greater flexibility under varying sampling conditions.

### 2.2. Entropy-Based Neural Network

The experiments described in Section 4.2.5 were systematically repeated, using the same neural network architecture and validation protocol. Incorporating entropy-related features produced performance that was comparable to SNP-only models, with only marginal differences observed in cross-validation and no consistent improvement in held-out test performance. The best-performing configuration corresponded to the global subject entropy setup, reaching a cross-validation mAUC of 0.9033. However, when evaluated on the held-out test set, generalization performance decreased to an AUC of 0.8741, suggesting that the entropy-enriched models may have increased model variance. Given the absence of consistent improvement in held-out test performance, entropy was not included in the final externally validated model.

### 2.3. Threshold-Based Risk Assessment

**Figure 1:**
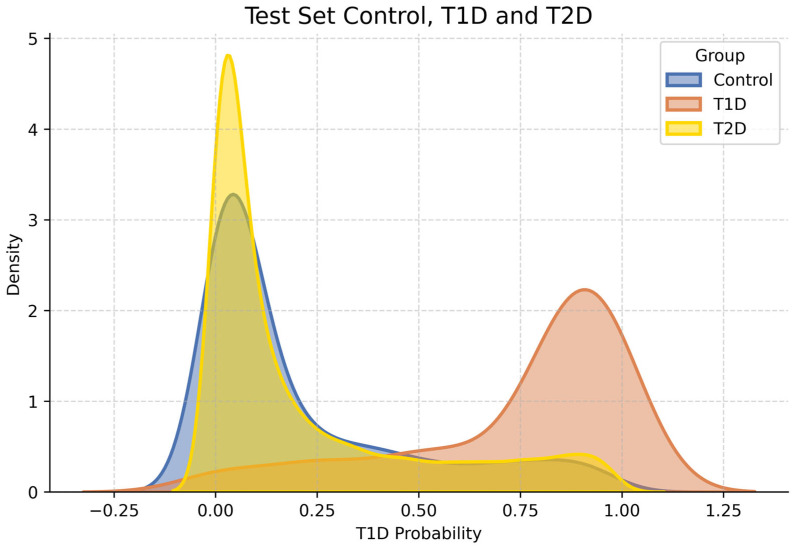
Risk-score distributions in the UK Biobank test set. Kernel Density Estimation curves depict predicted probabilities for T1D cases, non-diabetic controls, and a T2D auxiliary group. T2D subjects largely overlap with the control distribution, which is consistent with the model specificity for autoimmune (T1D) genetic architecture.

In order to facilitate the clinical interpretability of model outputs, the predicted probabilities for the test set were analyzed in a risk-stratified framework. Kernel Density Estimation (KDE) plots, shown in Figure 1, were generated to visualize the distribution of predicted probabilities for T1D cases and controls. These plots highlight the degree of overlap between both groups and the model’s ability to separate high-risk from low-risk individuals.

Based on the observed probability distribution of the cross-validated experiments and predefined interpretability objectives, five risk categories were defined: very low, low, average, high, and very high. The corresponding probability thresholds (0.1, 0.35, 0.65, and 0.9) were selected to represent progressively increasing levels of genetic risk while maintaining clinically meaningful trade-offs between sensitivity and specificity, and were not optimized on the test set. Lower thresholds prioritize high negative predictive values, which are suitable for screening contexts, whereas higher thresholds emphasize specificity and positive predictive values to identify individuals who are at substantially elevated genetic risk.

This threshold-based classification allows for a more intuitive interpretation of the predicted risk and facilitates potential clinical or epidemiological applications. Table 2 summarizes the distribution of subjects across the proposed risk categories in the test set, showing the percentage of cases and controls within each group. This threshold-based approach provides an interpretable framework to contextualize individual risk scores, illustrating the model’s discriminative capacity in a way that may be more actionable for risk communication or population-level stratification.

When applied to the subset of individuals with T2D, the model assigned probability values that were predominantly within the very low and low risk ranges, which was consistent with the distribution observed in the healthy controls, as can be seen in Table 2 and Figure 1. This result supports the notion that the genetic risk factors captured by the model are specific to autoimmune diabetes, and that T2D individuals, from a genomic standpoint, behave similarly to controls in the context of T1D risk prediction.

To further quantify the classifier performance under varying risk thresholds, sensitivity, specificity, and predictive values were calculated at the conventional 0.5 cut-off and the thresholds used in the risk assessment framework (0.1, 0.35, 0.65, 0.9). The results are summarized in Table 3.

As shown in Table 3, lowering the probability threshold increased the sensitivity at the expense of the specificity, whereas higher thresholds improved the positive predictive value (PPV) but reduced the sensitivity. At the upper end, the “very high” risk category achieved excellent specificity (97.3%) and the highest positive predictive value (61.9%), suggesting its potential for identifying individuals with a strong genetic predisposition to T1D. Conversely, those classified within the “very low” risk group exhibited a negative predictive value (NPV) of 98.9%, indicating that the model reliably excludes genetically low-risk profiles. It is important to note that these positive and negative predictive values were derived from the held-out test set, which preserved the true population prevalence of approximately 10% T1D cases. Under these conditions, PPV and NPV provide realistic estimates of how the model might perform in a representative European screening context. However, when applied to external datasets with different case–control ratios, these metrics become prevalence-dependent and should therefore be interpreted with caution. In such scenarios, AUC and sensitivity/specificity remain the most robust indicators of classifier performance.

Overall, these findings demonstrate that the neural network-derived probabilities can be translated into clinically interpretable risk categories, enabling both individualized assessment and population-level stratification. To further examine the generalizability of the model and evaluate the stability of these thresholds under different prevalence conditions, the same probability-based framework was subsequently applied to an external European cohort, as detailed in the following section.

### 2.4. Model Validation on an External European Cohort

To further evaluate the robustness and generalizability of the proposed neural network, an external validation was performed using an independent cohort provided by a collaborating German research group. This cohort presented an unbalanced case–control distribution, with a markedly higher T1D prevalence than the 10% ratio maintained in the UK Biobank test set. Consequently, while sensitivity and specificity remain valid indicators of classifier discrimination, PPV and NPV must be interpreted with caution, as they are directly influenced by prevalence. In this context, PPV and NPV are not fully comparable to those obtained in the UK Biobank sample, but can still offer qualitative insight into the model’s calibration under different epidemiological conditions. This independent validation cohort comprised 367 T1D cases and 123 controls. Within the T1D group, participants were further categorized according to their GADA status, a common immunological marker distinguishing autoimmune (GADA+) from non-autoimmune (GADA-) diabetes forms. GADA positivity reflects the presence of autoimmune β-cell destruction, whereas GADA-negative cases may correspond to atypical or mixed phenotypes. Among the T1D group, 295 individuals were GADA+ and 72 were GADA-. This stratification enabled us to assess whether the model differentially recognized autoimmune-driven genetic risks.

**Figure 2:**
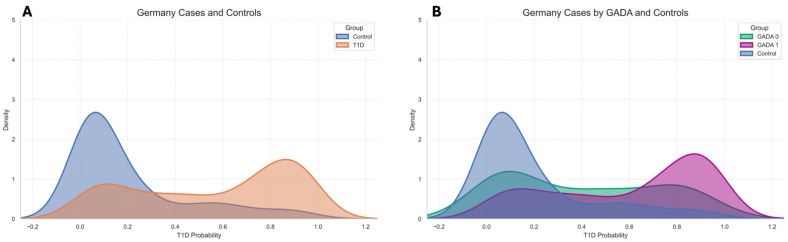
External validation in the external European (German) cohort and stratification by GADA status: (**A**) KDE curves of predicted probabilities for all T1D cases and controls show clear separation. (**B**) Within cases, KDE curves for GADA+ (GADA1) and GADA− (GADA0) illustrate the shift toward higher predicted risk among autoimmune (GADA+) subjects.

KDE plots (Figure 2) were generated to visualize the distribution of predicted probabilities in the GDS separately for cases and controls, and were further stratified by GADA, potentially reflecting distinct genetic architectures. To quantitatively evaluate the model performance in the external European cohort, sensitivity and specificity were computed at the same probability thresholds applied to the UK Biobank dataset (0.10, 0.35, 0.50, 0.65, and 0.90). For this analysis, only glutamic acid decarboxylase antibody-positive (GADA+) cases were compared against the controls, representing the autoimmune form of type 1 diabetes. These results are summarized in Table 4. Following the same methodology applied to the UK Biobank test set, five risk categories were defined, based on probability thresholds of 0.1, 0.35, 0.65, and 0.9, corresponding to very low, low, average, high, and very high risk levels. Table 5 summarizes the distribution of subjects across these categories for the external European validation cohort, showing the percentage of controls, GADA- cases, and GADA+ cases within each group.

#### Comparison with T1D GRS2

To contextualize the neural network’s performance, we computed the original T1D GRS2 on the German cohort. GRS2 achieved an AUC of 0.8334 (95% CI: 0.7938–0.8730) for case–control discrimination, which was slightly higher than the neural network (AUC 0.8086; 95% CI: 0.7657–0.8514). Stratification by GADA status revealed AUCs of 0.8715 (95% CI: 0.8348–0.9083) for GADA+ and 0.6771 (95% CI: 0.5961–0.7580) for GADA- individuals, compared with 0.8389 (95% CI: 0.7976–0.8801) for GADA+ and 0.6845 (95% CI: 0.6041–0.7649) for GADA-, respectively, for the neural network. Thus, both models performed comparably across all groups, with GRS2 showing marginally higher AUC values. A formal statistical comparison between AUCs was not performed, as the external validation analysis was intended to be descriptive, rather than a powered superiority assessment; interpretation is therefore guided by the reported confidence intervals. As summarized in Table 6, when using clinically relevant thresholds, the neural network preserved higher positive predictive values and stricter high-risk categorization, assigning fewer controls to the upper risk stratification range while maintaining comparable sensitivity. These findings indicate that although GRS2 offers slightly higher global and GADA+ AUC values, the neural network provides more selective high-risk identification and more selective high-risk categorization within the upper probability range.

Overall, the external validation supports the generalizability of the neural network classifier across independent European cohorts. Despite the higher T1D prevalence and the absence of 14 SNPs, the model preserved strong discriminative performance, with probability distributions clearly separating the cases from the controls. The slightly higher PPVs observed in the German cohort reflect the expected influence of prevalence on predictive metrics, rather than overfitting or loss of calibration. Importantly, similar trends across the GADA+ and GADA− subgroups indicate that the classifier captures a shared genetic signal underlying autoimmune susceptibility, while still recognizing variability among non-autoimmune cases. These results support the robustness of the probability-based risk framework within comparable European populations and provide a foundation for further validation in broader and multi-ancestry settings.

## 3. Discussion

This study demonstrates the feasibility and effectiveness of a neural network-driven approach to stratify genetic risk for T1D using a compact SNP panel and entropy-based features. The utility of using a neural network is their ability to model complex, nonlinear interactions between SNPs (epistasis), which are only partially captured by additive polygenic risk scores. In contrast to linear models that assume independent and additive SNP effects, multilayer neural architectures can learn higher-order dependencies and conditional relationships between loci, potentially reflecting the underlying immunogenetic architecture of type 1 diabetes better. We emphasize that this is particularly relevant in and around the HLA region, where epistatic combinations of alleles contribute disproportionately to disease risk, and where nonlinear modeling may recover a signal that is not exploited by standard PRS frameworks. This framework outputs the individualized probability estimates of T1D, rather than arbitrary risk scores, which facilitates direct calibration to clinically meaningful thresholds (e.g., for newborn screening or reclassification of atypical diabetes). Probabilistic outputs allow for explicit control of sensitivity/specificity trade-offs, support decision-curve-type analyses, and are naturally integrable into Bayesian or risk-communication frameworks in clinical practice

Validation in the external German cohort supports transferability across independent European cohorts and suggests that model performance is not specific to a single dataset within populations of similar genetic ancestry. However, because both validation cohorts consisted of individuals of European ancestry, these findings should not be interpreted as evidence of generalizability across diverse ancestral populations. Genetic risk architecture varies across populations, and performance may differ in non-European cohorts. The high AUC values achieved with a compact 67-SNP panel are consistent with the known genetic architecture of T1D, in which high-effect loci, particularly within the HLA region, contribute substantially to risk discrimination. The SNP set used here derives from the validated GRS2 framework. To reduce the overfitting risk, model development incorporated dropout regularization, stratified cross-validation within the training data, and evaluation on both a held-out test set and an independent external cohort. The consistency of discrimination across these stages supports the robustness of the reported performance. When compared directly with the recently described T1D GRS2, the neural network demonstrated broadly comparable discriminative performance in the German cohort. In GADA- individuals, where genetic signal is expected to be weaker and phenotypic heterogeneity greater, both methods showed reduced discrimination. Despite slightly lower AUC values, the neural network provided clearer stratification at high-risk thresholds, with fewer controls entering upper probability categories and higher PPVs at clinically actionable cut-offs. This suggests that nonlinear architectures may capture multi-locus interactions that improve the calibration of the highest-risk profiles, complementing the strong linear performance of the GRS2 model. Overall, the results support the neural network as a competitive and flexible alternative to GRS2. The proposed approach demonstrated more selective upper-tail risk assignment and higher positive predictive values at clinically relevant thresholds, suggesting potential advantages in risk stratification, rather than overall discrimination.

Importantly, the added value of our approach does not primarily reside in uniformly higher performance, but in its probabilistic behavior and structural flexibility. The model enables direct optimization of nonlinear interactions among loci without predefined weighting schemes and produces continuous probability estimates that can be adapted to different clinical contexts. Furthermore, the architecture facilitates future extensions beyond SNP-only models. In this sense, the neural network framework should be interpreted as a flexible modeling platform, rather than a replacement that is strictly judged by performance superiority.

Together, these findings strengthen confidence in the use of neural networks for polygenic risk modeling and provide further evidence that such architectures can generalize across independent European cohorts with comparable genetic architectures and disease prevalences. Consistently with the prior research [12], our results reaffirm the utility of both raw and β-weighted allele encoding for polygenic modeling. Although β-weighted encodings offer a theoretically improved signal-to-noise ratio by emphasizing SNPs with stronger associations, the performance difference between encoding strategies was minimal. This may suggest that simple allele-count encoding captures most of the predictive information. The 67-SNP panel evaluated here includes several highly informative variants, particularly within the HLA region, which contribute substantially to discrimination. In relatively compact panels dominated by high-effect loci, externally derived β-weighting may offer limited incremental benefit, especially when nonlinear models can implicitly learn differential feature weighting during training. Additionally, the relative genetic homogeneity of the European ancestry cohorts may reduce the variability in effect-size transferability. Importantly, this finding should not be interpreted as evidence that β-weighting lacks value in broader polygenic architectures or multi-ancestry contexts, where large numbers of low-effect variants and effect-size heterogeneity may alter the relative contribution of weighted scoring approaches.

The use of controlled class-imbalanced training proved beneficial in maximizing discriminative ability. As described in Section 4.2.2, imbalance was handled through random undersampling of controls to construct nested case–control ratios, while class-weighted loss functions were evaluated but did not produce meaningful performance gains. Moderate imbalance (particularly the 1:3 ratio) consistently yielded the highest cross-validation AUCs across encoding schemes.

From a methodological perspective, undersampling may increase variance due to the reduced training data size, whereas extreme imbalance may bias the model toward the majority class and impair minority-class discrimination. In our experiments, moderate imbalance appeared to provide a favorable trade-off between learning stability and case discrimination. Nevertheless, undersampling-based strategies may influence the probability calibration, and future work incorporating formal calibration analyses or alternative reweighting strategies could further clarify these effects. While HWE-based imputation is reasonable for rare missing data and non-selected SNPs, it may introduce bias at immune-related loci like HLA, where deviations from HWE are frequent due to evolutionary selection and disease association; future work will employ LD-based imputation (e.g., IMPUTE2 or Michigan Imputation Server) to mitigate this.

Entropy, used here as an information-theoretic descriptor of genotype complexity, was explored as a secondary feature to assess whether aggregate deviation from population allele frequencies could provide complementary signal beyond individual SNP effects. In cross-validation experiments, entropy showed modest and somewhat variable contributions to performance, and it ranked among influential features in certain configurations (Supplementary Information). However, its impact on held-out test discrimination was limited and did not consistently enhance the generalization. These findings suggest that entropy may capture aspects of genotype distribution that are not fully reflected by individual SNP inputs, potentially representing aggregate deviation from population norms. Nevertheless, its contribution appears to be context-dependent, and it should be interpreted as exploratory, rather than as a validated improvement to the predictive framework. Its usefulness may vary, depending on model architecture, regularization strategy, and the size and composition of the genetic panel, particularly in compact SNP frameworks dominated by high-effect loci.

Several limitations must be acknowledged. First, the external validation cohort was relatively small and imbalanced, particularly within subgroup analyses. Consequently, confidence intervals and discrimination estimates should be interpreted with appropriate caution. The model was trained and validated exclusively in cohorts of European ancestry, minimizing confounding due to population stratification but limiting immediate generalizability to other ethnic groups. Accordingly, the discrimination metrics reported here should be interpreted as ancestry-specific performance estimates, rather than universally transferable measures of predictive accuracy. Thus, validation across other ancestry-derived cohort sets is mandatory to evaluate the performance of this model in other ethnicities. The reduced performance of European-derived GRSs in African and admixed populations has been well-documented [11,13], underscoring the need for ancestry-aware calibration and model adaptation. Future work should extend this framework to multi-ancestry datasets, potentially leveraging transfer learning or ancestry-informative markers to improve cross-population performance. Second, while our model did not explicitly model HLA haplotype interactions, it included SNPs that were strongly associated with key HLA class II risk alleles. These SNPs were in linkage disequilibrium with HLA-DR and HLA-DQ haplotypes that were known to confer T1D risk. Therefore, our approach may have effectively captured much of the relevant HLA signal through these proxy markers. Nevertheless, future work incorporating explicit HLA haplotyping interaction terms could further enhance the model accuracy and interpretability, especially in diverse populations or cases with ambiguous or atypical serological profiles. Proxy HLA SNPs facilitate practical T1D risk prediction but may underperform in non-DR3/DR4 cases or diverse ancestries, due to LD variability and incomplete haplotype coverage; explicit HLA typing is preferable for precise stratification in such contexts. Additionally, formal calibration analyses were not performed in the present study. While the model provides probabilistic outputs and threshold-based risk categories, future work should formally evaluate calibration properties to further support clinical translation. Finally, while the model achieved strong discrimination, translating polygenic predictions into clinical decision-making will require further validation in prospective and pediatric cohorts, where pre-symptomatic identification is the most impactful. Integration of genomic risk with clinical and immunological markers may enhance the prediction of disease progression and support personalized prevention strategies.

## 4. Materials and Methods

### 4.1. Dataset

For this study, we used genotype data extracted from the UK Biobank [16] under the project approval ID: 93016. Specifically, we analyzed data from the imputation folder, which contains the UK Biobank Affymetrix Axiom Array (Santa Clara, CA, USA) data and the imputed variants provided by the UK Biobank.

As of 17 February 2025, we have selected 546 T1D based on the inclusion criteria defined by Sharp et al. [12]: (a) initial clinical diagnosis of diabetes at ≤20 years of age; (b) on insulin within 1 year from the time of diagnosis; (c) still on insulin at the time of recruitment; (d) not using oral antihyperglycemic agents; and (e) did not ever self-report as having T2D. Additionally, 11,363 controls were selected according to the following criteria: (f) no diagnosis of diabetes made by a doctor; (g) no self-reported non-cancer illness codes related to any form of diabetes, including type 1, type 2, gestational diabetes, diabetes insipidus, or endocrine/diabetes conditions; (h) no insulin therapy initiated within one year of diabetes diagnosis; and (i) no hospital diagnosis records with ICD-10 or ICD-9 codes related to diabetes (specific codes excluded as per 56 ICD-10 and 30 ICD-9 values). The final dataset consisted of 11,909 individuals, which was split into a training set (80%) and a test set (20%) while maintaining a balanced distribution of cases and controls (Supplementary Figure S2).

In addition to the previous groups, 29,849 individuals diagnosed with T2D were also included as an auxiliary control group and selected according to the following criteria: (j) ICD-10 diagnostic field p41270 contained the code E11.9 (type 2 diabetes mellitus without complications); (k) individuals with any E10-prefix code (type 1 diabetes) were excluded; (l) self-reported ethnicity (penero21000_i0) was “British” or “Irish”; and (m) genetic data were available. This decision was made to evaluate whether the proposed model, trained to predict T1D, distinguishes autoimmune from non-autoimmune diabetes forms. From a genetic perspective, T2D is not expected to share the same genetic risk architecture as T1D. Therefore, their inclusion serves as an additional negative control, allowing the assessment of the model’s ability to differentiate autoimmune diabetes from other metabolic forms of diabetes.

From these individuals, we selected 67 SNPs that were previously identified as being associated with T1D in [12], as shown in Table 7.

#### Missing Data Treatment

Among the 11,909 subjects included in this study, 399 individuals (222 cases and 177 controls) presented with missing values across the 67 analyzed SNPs. To address this issue, the missing genotypes (Supplementary Table S1) were imputed under the assumption of the Hardy–Weinberg equilibrium, using allele frequencies estimated from a representative subsample of the population that preserved a T1D prevalence of 10%. Because this reference population predominantly reflects non-diabetic individuals, frequency-based imputation may introduce a slight bias toward control-like genotype distributions. Importantly, this bias would be expected to attenuate genetic risk among true T1D cases, rather than inflate discrimination performance. A fixed T1D prevalence of 10% was used to estimate the PPV and NPV, as is conventional for assessing diagnostic performance in high-risk populations (e.g., first-degree relatives or autoantibody-positive individuals), where disease enrichment is typical and the general population prevalence (~0.3%) would unrealistically inflate NPV while understating clinical utility.

Imputation was performed independently of case–control labels and was not stratified by disease status. This strategy was adopted to reflect realistic clinical deployment, where the disease status is unknown at the time of genotype processing, and to prevent circularity or information leakage during model development and validation. Genotype imputation for the missing data was performed assuming HWE, as is commonly implemented in GWAS pipelines.

### 4.2. Study Design

To leverage artificial intelligence for genomic modeling, we implemented a feed-forward neural network architecture optimized for binary classification. Neural networks are particularly suitable for capturing complex, non-linear relationships among genetic variants and can generalize well to unseen data when appropriately regularized.

#### 4.2.1. Data Encoding

Accurate SNP data encoding is crucial for model performance. Therefore, two encoding strategies were evaluated: (1) risk allele count—number of risk alleles present (0, 1, or 2) and (2) weighted allele count—number of risk alleles multiplied by the associated β coefficient [12] (0, 1, or 2 × β). The performance of both encoding schemes was compared to determine the most suitable approach.

The β coefficients reported in Table 7 were taken from Sharp et al. [12] and are presented to document the established GRS2 panel and to define the optional β-weighted encoding. They were not used to define interaction or dominance terms, and no explicit epistatic features were used.

#### 4.2.2. Data Imbalance

As noted in Section 4.1, our dataset is highly imbalanced, with over 95% of the subjects being controls. Class imbalance can negatively affect model performance, particularly by biasing predictions toward the majority class. To mitigate this issue, several nested case-to-control ratios (1:1, 1:2 and 1:3) were tested in order to determine the proportion that yielded the most effective case–control discrimination using neural networks.

For each ratio, the controls were randomly undersampled while retaining all available T1D cases. The evaluated subsets were nested, such that the 1:1 dataset was fully contained within the 1:2 dataset, which in turn was contained within the complete 1:3 dataset. No oversampling of cases was performed.

Class-weighted loss functions were also evaluated in preliminary experiments (Figure S1), but did not yield meaningfully. Therefore, undersampling was selected as the primary imbalance-handling strategy to allow for systematic comparison across ratios.

For each ratio-specific dataset, stratified five-fold cross-validation was performed to preserve the case–control proportion within each fold. No additional resampling was conducted inside the folds. For the held-out test set, a fixed 1:9 ratio was employed, reflecting the approximate 10% T1D prevalence in European populations, and this ratio remained unchanged throughout all UK Biobank experiments. The test set was not used for model training or hyperparameter selection.

#### 4.2.3. Neural Network Architecture and Regularization

The implemented model followed a feed-forward neural network architecture composed of an input layer, three fully connected hidden layers and a single output layer for binary classification. The hidden layers contained progressively fewer neurons (256, 128, and 64) to encourage hierarchical feature abstraction and reduce overfitting. Each hidden layer used the Rectified Linear Unit (ReLU) activation function, which facilitates efficient learning by mitigating vanishing gradient issues and enabling the network to capture non-linear relationships among genetic variants. To prevent overfitting and enhance generalization, dropout layers were interleaved between the dense layers. Dropout [17] is a regularization technique in which a random subset of neurons is temporarily deactivated during training. This approach prevents overfitting by reducing reliance on specific nodes or patterns, thereby improving model robustness on unseen data. In this model, the dropout rates decreased progressively across layers (from 40% to 20%) to balance the regularization strength with the decreasing representational capacity of deep layers. The final output layer consisted of a single neuron with a sigmoid activation function, producing a probability value between 0 and 1, corresponding to the likelihood of an individual being classified as a T1D case.

Model training was performed using binary cross-entropy as the loss function. Hyperparameters were optimized through grid search within the training set, using stratified five-fold cross-validation. The grid search explored different optimizers (stochastic gradient descent (SGD) and Adam) and learning rates (0.1, 0.01, 0.001).

SGD with a learning rate of 0.001 provided the most stable cross-validation performance and was selected for the final model. The batch size was fixed at 32. The maximum number of training epochs was set to 1000. However, early stopping was implemented to prevent overfitting by monitoring validation loss within each cross-validation fold, with restoration of the best-performing weights. Network weights were initialized using Glorot uniform initialization.

#### 4.2.4. Model Validation

For each combination of class ratio and SNP encoding method, we have conducted a grid search with 5-fold cross-validation on the training set. The best-performing hyperparameter configuration, as determined via cross-validation, was used to retrain the model over the whole training set. This model was used to perform validation on the test set. Additionally, to assess the reproducibility and external generalization, the trained neural network was applied to the independent German Diabetes Study (GDS) cohort without retraining, recalibration, or modification of learned weights or probability thresholds. The GDS recruits individuals with recent-onset diabetes within the first year of diagnosis and matched healthy controls [18]. A subset of the study comprising persons with T1D and healthy controls at the Düsseldorf site has been genotyped. Participants diagnosed with T1D based on ADA criteria [19] were selected as cases and diabetes-free controls as the controls. GADA were measured using a radioimmunoassay (Medipan GmbH, Dahlewitz, Germany). Genotyping was performed on a total of 367 individuals with T1D and 123 control subjects. Genotype data were subsequently imputed on the Michigan Imputation Server (v2.0.0-beta3), using Minimac4 (v4.1.6) and the TOPMed r3 reference panel. This external validation ensured that model performance was not dataset-specific and provided a more reliable estimate of its predictive capacity across different populations.

Importantly, among the 67 SNPs included in the neural network-driven PRS model, 14 were missing in the external European dataset, because of the technology used for genotype data generation. Following the procedure described in the Section Missing Data Treatment, these missing variants were imputed under the assumption of the Hardy–Weinberg equilibrium, using allele frequencies estimated from the corresponding reference population.

For contextual benchmarking, we additionally evaluated the widely used T1D GRS2 [12] genetic risk score, which is derived from the same 67 SNPs included in our neural network model. GRS2 was computed in the external German cohort, using a public release. Its performance was assessed using AUC and the same threshold- and category-based metrics applied to the neural network, allowing for a direct comparison of discriminative ability and risk stratification. AUC confidence intervals (95%) were computed using DeLong’s nonparametric method to account for sampling variability in both internal and external validation cohorts.

#### 4.2.5. Entropy Exploratory Experiments

To further explore the potential contribution of information-theoretic descriptors in genetic modeling, we have incorporated the variable entropy as an additional feature in our neural network model. Entropy provides a quantitative measure of uncertainty or variability within a subject’s genotype, reflecting the degree of deviation from the population reference frequencies. For each individual, entropy was computed using the allele frequencies derived from the reference population described in Section Missing Data Treatment. Specifically, for every SNP *i,* the frequency of each genotype *p_i_* was obtained from the reference population cohort, and the entropy term was computed as follows:

$$H\;=\;-\sum_{i\;=\;1}^{67}p_{i}\;\cdot \;{log}_{2}({\;p}_{i})$$

where *p_i_* represents the frequency of the subject’s genotype at the *i*-th SNP within the reference population. This formulation captures the information content of an individual’s genotype, relative to population expectations. Individuals carrying rare genotypes exhibit higher entropy values, reflecting greater deviation from the expected allelic distribution.

Several configurations were tested to assess the relevance of this variable: (1) global subject entropy, computed across all 67 SNPs; (2) per-chromosome entropy, where entropy values were independently calculated for SNPs belonging to each chromosome; and (3) combined entropy features, where both global and per-chromosome entropy values were concatenated to form an extended feature set.

Each of these variants was introduced as an additional input feature to the neural network described in Section 4.2.3, and all experiments were repeated under identical training and validation conditions to evaluate whether entropy contributed to improved discriminative performance in T1D classification.

## 5. Conclusions

Our findings reinforce the clinical value of neural network-driven genetic risk scores as a practical tool to support more accurate diabetes subtype classification when clinical features are ambiguous. By delivering probabilistic risk estimates that align with clinically intuitive thresholds, this model can help to distinguish autoimmune from non-autoimmune diabetes at presentation, particularly in youth and adults who may otherwise be misdiagnosed as T2D. Integrating this approach with existing clinical and immunological markers could enable risk-adapted follow-up strategies, early referral for immunomodulatory therapy, and optimized long-term management. The neural network framework is inherently scalable and adaptable: once implemented, the same architecture can be retrained or fine-tuned as new cohorts, ancestries, or feature types (e.g., epigenetic marks, transcriptomic readouts, or updated SNP panels) become available. Such models can be periodically recalibrated to maintain performance across evolving clinical populations and genotyping platforms, and the modular design of our pipeline facilitates integration into existing precision-medicine workflows [20]. This contrasts with static PRS formulas that are less flexible when transported across ancestries or when the underlying SNP set changes.

Given its compact SNP panel and strong generalizability, this framework may represent a promising complementary tool into digital clinical-decision tools or population-based screening programs. Ultimately, such genomics-assisted classification may improve treatment precision, preserve β-cell function, and reduce the burden associated with delayed T1D diagnosis.

## Figures and Tables

**Table 1:** Results of the neural network experimentation results from cross-validation setup and its generalization with test set. Best values are in bold.

Data Ratio	Data Encoding	CV mAUC
1:1	0/1/2	0.8845
	0/1/2 × β	0.8824
1:2	0/1/2	0.8969
	0/1/2 × β	0.8963
1:3	0/1/2	**0.9027**
	0/1/2 × β	0.8263
All available	0/1/2	0.8976
	0/1/2 × β	0.8986

**Table 2:** Distribution of proposed risk categories among test set individuals.

Risk	Control (%)	T1D (%)	T2D (%)
Very low	54.54	5.50	53.56
Low	22.43	7.34	22.97
Average	11.52	13.76	10.89
High	8.77	33.03	9.12
Very high	2.75	40.37	3.46

**Table 3:** Performance metrics of the neural network classifier at selected probability thresholds, using cases and controls from the test set. PPV: positive predicted value and NPV: negative predicted value.

Threshold	Sensitivity	Specificity	PPV	NPV
0.1 (Very low)	0.9450	0.5454	0.1876	0.9889
0.35 (Low)	0.8716	0.7696	0.2960	0.9818
0.5 (Average)	0.8165	0.8379	0.3589	0.9762
0.65 (High)	0.7339	0.8848	0.4145	0.9677
0.9 (Very high)	0.4037	0.9725	0.6197	0.9362

**Table 4:** Performance metrics of the neural network classifier at selected probability thresholds over GADA+ external European cohort. Positive predictive value (PPV) and negative predictive value (NPV) included.

Threshold	Sensitivity	Specificity	PPV	NPV
0.1 (Very low)	0.8983	0.5366	0.8230	0.6875
0.35 (Low)	0.7288	0.8049	0.8996	0.5531
0.5 (Average)	0.6305	0.8455	0.9073	0.4883
0.65 (High)	0.5661	0.9187	0.9435	0.4689
0.9 (Very high)	0.2271	0.9756	0.9571	0.3448

**Table 5:** Distribution of proposed risk categories among the external European cohort by GADA status and combined.

Risk	GADA+ (%)	GADA− (%)	T1D (%)	Control (%)
Very low	10.17	25.00	13.08	53.66
Low	16.95	25.00	18.53	26.83
Average	16.27	19.44	16.89	11.38
High	33.90	26.39	32.43	5.69
Very high	22.71	4.17	19.07	2.44

**Table 6:** T1D GRS2 (normalized) performance metrics at selected probability thresholds over GADA+ external European cohort. Positive predictive value (PPV) and negative predictive value (NPV) included.

Threshold	Sensitivity	Specificity	PPV	NPV
0.1 (Very low)	1.0000	0.0163	0.7091	1.0000
0.35 (Low)	0.9831	0.3496	0.7838	0.8958
0.5 (Average)	0.8847	0.6341	0.8529	0.6964
0.65 (High)	0.6373	0.8780	0.9261	0.5023
0.9 (Very high)	0.0814	1.0000	1.0000	0.3122

**Table 7:** List of the 67 SNPs (GRCh37) used in this study, including the risk allele and corresponding β coefficient from Sharp et al. [12]. These SNPs were selected for their established association with type 1 diabetes and include variants linked to both HLA and non-HLA loci. β: beta value for each SNP.

dbSNP ID	Risk Allele	β	Chromosome	Position	Genes
rs9275490	G	2.08	6	32673385	
rs540653847	I	1.78	6	31274794	*LOC112267902*
rs9271346	T	1.69	6	32583468	
rs17843689	T	1.39	6	32622047	*HLA-DQA1*
rs9273369	C	1.26	6	32626484	*HLA-DQB1-AS1*
rs116522341	C	1.24	6	32367697	*BTNL2*, *TSBP1-AS1*
rs1281934	G	0.9	6	32584382	
rs2567287	A	0.84	6	33049185	*HLA-DPA1*, *HLA-DPB1*
rs3842753	G	0.83	11	2181060	*INS*, *INS-IGF2*
rs75658393	T	0.81	6	32395517	
rs72848653	T	0.78	6	29836602	
rs144530872	A	0.74	6	29863887	
rs9269173	A	0.67	6	32447188	
rs2476601	A	0.64	1	114377568	*PTPN22*, *AP4B1-AS1*
rs9500974	T	0.63	6	29728253	
rs17211699	T	0.48	6	32626037	*HLA-DQB1-AS1*
rs12189871	T	0.45	6	31251924	
rs12153924	A	0.44	6	29914089	*HLA-A*, *LOC124901298*
rs371250843	D	0.39	6	31324737	*HLA-B*, *MIR6891*
rs9259118	T	0.31	6	29850909	
rs2289702	C	0.28	15	79237293	*CTSH*
rs4948088	C	0.26	7	51027194	
rs653178	C	0.26	12	112007756	*ATXN2*
rs559242105	I	0.24	6	33071028	
rs4759229	A	0.22	12	56474480	*ERBB3*
rs9924471	A	0.22	16	28591530	*SGF29*
rs1893217	G	0.19	18	12809340	*PTPN2*
rs72928038	A	0.18	6	90976768	*BACH2*
rs60888743	A	0.18	10	90051317	*RNLS*, *LOC101929727*
rs11170466	T	0.17	12	53585859	*ITGB7*, *ZNF740*
rs9981624	C	0.17	21	43825722	*UBASH3A*
rs9388489	A	0.16	6	126698719	*CENPW*
rs425105	T	0.15	19	47208481	*PRKD2*
rs5763779	A	0.15	22	30504652	*HORMAD2*
rs72727394	T	0.14	15	38847022	*RASGRP1*
rs17388568	A	0.12	4	123329362	*ADAD1*
rs1615504	T	0.12	18	67526644	*CD226*
rs6476839	T	0.11	9	4290823	*GLIS3*
rs9585056	C	0.11	13	100081766	
rs2281808	C	0.1	20	1610551	*SIRPG*
rs229541	A	0.1	22	37591318	*C1QTNF6*
rs9469200	C	−0.03	6	32603212	*HLA-DQA1*, *LOC124901301*
rs1738074	T	−0.08	6	159465977	*TAGAP*, *TAGAP-AS1*
rs56994090	C	−0.13	14	101306447	*MEG3*
rs10492166	A	−0.14	12	9885999	*CLECL1*
rs3024505	A	−0.15	1	206939904	
rs2111485	A	−0.16	2	163110536	*LOC105373724*
rs3087243	A	−0.17	2	204738919	*CTLA4*
rs17214657	C	−0.19	6	33047173	*HLA-DPA1*, *HLA-DPB1*
rs12708716	G	−0.19	16	11179873	*CLEC16A*
rs144309607	T	−0.4	19	10492274	*TYK2*
rs10947332	A	−0.46	6	32677440	
rs61839660	T	−0.48	10	6094697	*IL2RA*
rs9378176	G	−0.49	6	33049309	*HLA-DPA1*, *HLA-DPB1*
rs2524277	A	−0.6	6	31407579	*LINC01149*
rs1281935	T	−0.63	6	32583820	
rs62406889	T	−0.65	6	32672214	
rs6934289	C	−0.68	6	33044956	*HLA-DPA1*, *HLA-DPB1*
rs41295121	T	−0.71	10	6129643	*RBM17*
rs28746898	G	−0.76	6	32648594	
rs16899379	A	−0.83	6	31343267	
rs12527228	T	−0.89	6	32679992	
rs149663102	D	−0.94	6	31344183	
rs1794265	G	−1.31	6	32674737	
rs9405117	A	−1.43	6	32602751	*LOC124901301*
rs16822632	A	−2.21	6	32411712	*HLA-DRA*
rs117806464	A	−2.41	6	32626447	*HLA-DQB1-AS1*

## Data Availability

All data for this project were obtained from the UK Biobank through application number 93016. The implementation used can be found at https://github.com/sethsh7/PRSedm.git. Accessed: 1 November 2025.

## Supplementary Materials

The following supporting information can be downloaded at: https://www.mdpi.com/article/10.3390/ijms27072966/s1.

## Abbreviations

The following abbreviations are used in this manuscript:

AA	African Ancestry
AUC	Area Under the Receiver Operating Characteristic Curve
EA	European Ancestry
GADA	Glutamic Acid Decarboxylase Antibody
GRS	Genetic Risk Score
GWAS	Genome-Wide Association Studies
HLA	Human Leukocyte Antigen
KDE	Kernel Density Estimation
NPV	Negative Predictive Value
PPV	Positive Predictive Value
PRS	Polygenetic Risk Score
ReLU	Rectified Linear Unit
SGD	Stochastic Gradient Descent
SNP	Single Nucleotide Polymorphism
T1D	Type 1 Diabetes
T2D	Type 2 Diabetes
