# Supplementary material for: Enhancing Type 1 Diabetes Polygenic Risk Prediction Through Neural Networks and Entropy-Derived Insights
Source: Int J Mol Sci. 2026 Mar 25;27(7):2966. doi: 10.3390/ijms27072966 (PMC13073193; doi:10.3390/ijms27072966)
Supplement: Supplementary file 1 [file ijms-27-02966-s001.zip › ijms-4151766-supplementary.pdf]

Supplementary Material: *Enhancing Type 1 Diabetes Polygenic Risk Prediction through Neural Networks and Entropy-Derived Insights*

Supplementary Figure S1. Preliminary Random Forest Experiments Supporting Entropy Feature Inclusion

During the initial phase of model exploration, a Random Forest classifier was implemented to assess feature relevance across the 67 T1D-associated SNPs and the entropy-derived variables described in Section 2.2.5.

The model was trained on the same UK Biobank dataset used for the neural network experiments, employing 10-fold cross-validation and balanced class sampling to mitigate the effect of case–control imbalance.

Feature importance was computed using the mean decrease in Gini impurity across all trees.

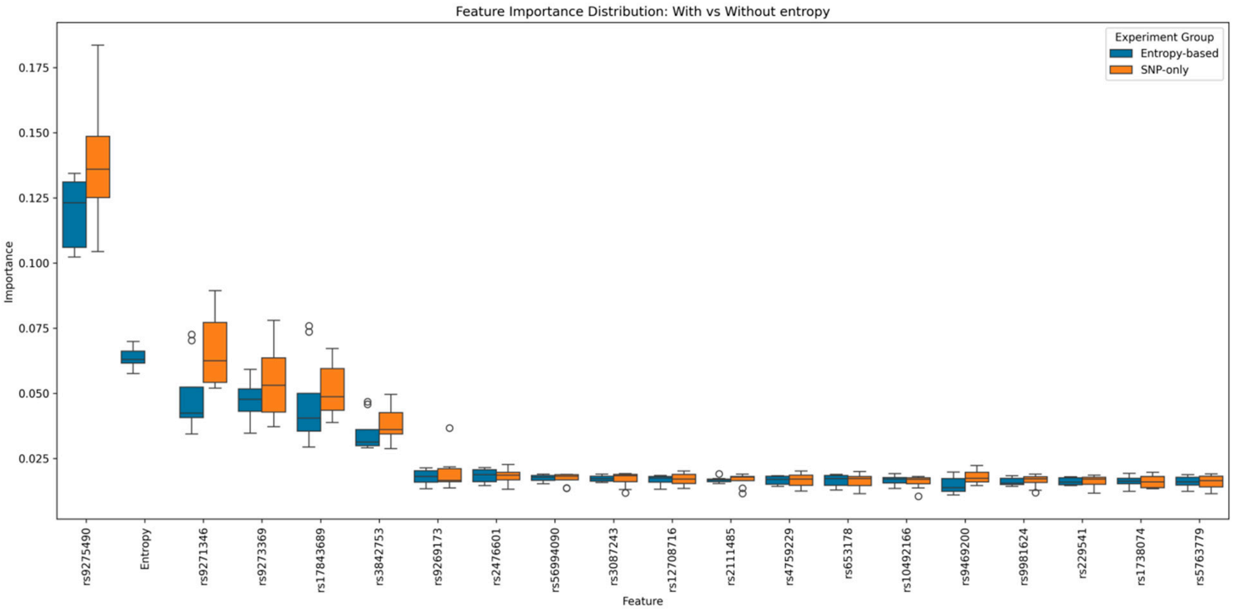

Figure S1. *Feature importance distribution in preliminary Random Forest models with and without entropy inclusion. Boxplot display the top 20 ranked features according to mean decrease in Gini impurity across a 5-fold cross-validation run. The comparison between the SNP-only model (orange) and the entropy-based model (blue) shows that the global genotype entropy feature consistently ranked among the top five predictors, together with well-established T1D-associated loci such as rs9275490, rs9271346, and rs3842753. These results support the inclusion of entropy-derived descriptors as complementary genomic features in subsequent neural network models.*

The top 20 features ranked by importance are shown in Figure S1, where the global genotype entropy variable consistently appeared among the top five predictors. These results motivated the inclusion of entropy-derived metrics in the subsequent neural network models.

While its contribution in the final architecture was modest, its early relevance in the Random Forest analysis suggests that entropy captures complementary information reflecting global genotype variability, which may enhance prediction under alternative modeling strategies.

Supplementary Figure S2. Study workflow and cohort overview for the neural network-based T1D polygenic risk model

Figure S2. Schematic diagram illustrating the dataset partitioning from the UK Biobank (UKB; n = 11,909 total: 546 T1D cases, 11,363 controls; split 80:20 into training [n = 1109 cases, 9090 controls] and test [n = 437 cases, 2273 controls] sets), with auxiliary T2D controls (n = 29,849). The model inputs comprise 67 T1D-associated SNPs and entropy and employs 5-fold cross-validation on the training set. Ex-ternal validation was performed on the German Diabetes Center (DDZ) cohort (n = 490: 367 T1D cases [295 GADA+, 72 GADA−], 123 controls), without retraining. Arrows denote data flow; sample sizes are indicated for key subsets.

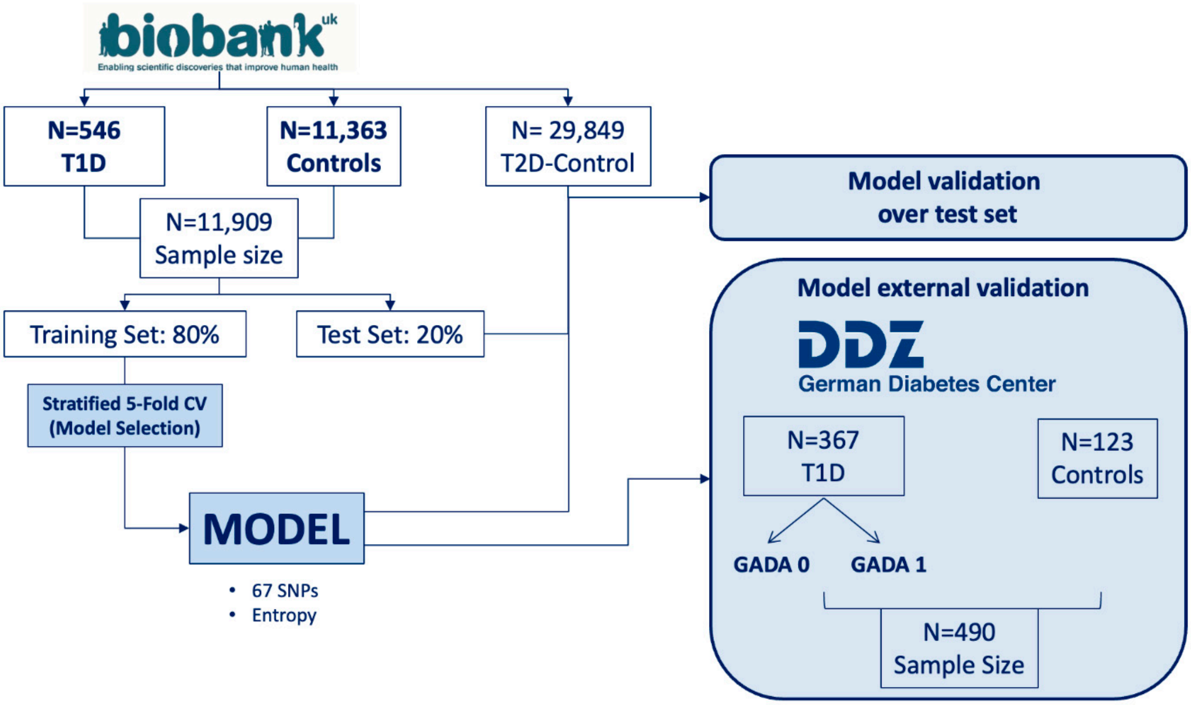

Supplementary Table 1. Percentage of missing genotype calls for the 67 SNPs (GRCh37) in the UK Biobank and external German validation cohorts prior to imputation

Supplementary table 1. Percentage of missing genotype calls for the 67 SNPs (GRCh37) in the UK Biobank and external German validation cohorts prior to imputation. Missingness values represent the proportion of individuals with unavailable genotype data for each SNP within the corresponding cohort. SNPs with 100% missingness in the German cohort indicate variants not directly available in the genotyping platform and subsequently imputed as described in Section 2.2.

dbSNP ID (GRCh37)	UKBioBank Missingness (%)	Germany Cohort Missingness (%)
rs9275490	0.03%	0.00%
rs540653847	0.13%	100%
rs9271346	0.03%	0.00%
rs17843689	0.42%	100%
rs9273369	0.00%	0.00%
rs116522341	2.28%	0.00%
rs1281934	4.41%	100%
rs2567287	0.00%	0.00%
rs3842753	1.36%	4.49%
rs75658393	0.01%	0.00%
rs72848653	0.08%	0.00%
rs144530872	0.37%	100%
rs9269173	1.96%	100%
rs2476601	0.00%	0.00%
rs9500974	0.02%	0.00%
rs17211699	0.47%	100%
rs12189871	0.00%	0.00%
rs12153924	0.36%	100%
rs371250843	0.03%	100%
rs9259118	0.06%	100%
rs2289702	0.39%	3.06%
rs4948088	0.00%	0.20%
rs653178	0.00%	0.20%
rs559242105	10.66%	100%
rs4759229	0.34%	5.10%
rs9924471	0.59%	16.94%
rs1893217	0.00%	0.00%
rs72928038	0.00%	0.00%
rs60888743	0.65%	1.43%
rs11170466	0.08%	1.63%
rs9981624	0.39%	8.57%
rs9388489	0.00%	0.00%
rs425105	0.00%	0.00%
rs5763779	1.08%	2.24%
rs72727394	0.83%	0.00%
rs17388568	0.00%	0.00%
rs1615504	0.06%	5.31%
rs6476839	0.37%	1.02%
rs9585056	0.00%	2.04%
rs2281808	0.00%	0.41%
rs229541	0.00%	0.20%
rs9469200	0.58%	0.00%

rs1738074	0.00%	0.20%
rs56994090	0.61%	27.96%
rs10492166	0.83%	1.22%
rs3024505	0.00%	0.00%
rs2111485	0.00%	0.20%
rs3087243	0.00%	0.00%
rs17214657	0.03%	0.00%
rs12708716	0.00%	0.00%
rs144309607	1.28%	100%
rs10947332	0.00%	0.00%
rs61839660	0.00%	0.00%
rs9378176	0.00%	0.00%
rs2524277	0.00%	0.00%
rs1281935	7.57%	1.22%
rs62406889	0.79%	0.41%
rs6934289	0.24%	0.20%
rs41295121	0.31%	0.41%
rs28746898	0.76%	100%
rs16899379	0.27%	0.00%
rs12527228	0.23%	100%
rs149663102	0.03%	0.00%
rs1794265	0.03%	100%
rs9405117	0.00%	0.00%
rs16822632	0.00%	0.00%
rs117806464	0.00%	0.00%
